# IMPACT OF MULTIDISCIPLINARY REHABILITATION ON FUNCTIONING AND QUALITY OF LIFE IN STROKE SURVIVORS: A LONGITUDINAL RETROSPECTIVE ANALYSIS

**DOI:** 10.2340/jrm.v58.42921

**Published:** 2026-01-14

**Authors:** Aet RISTMÄGI, Hannu HEIKKILÄ, Olavi AIRAKSINEN

**Affiliations:** Department of physical medicine and rehabilitation, Kuopio University hospital, Finland

**Keywords:** stroke, rehabilitation, health-related quality of life, EQ-5D, WHODAS 2.0, mRS, multidisciplinary care

## Abstract

**Objective:**

This study investigates the impact of different rehabilitation approaches on functional recovery and health-related quality of life (HRQoL) in stroke patients.

**Design:**

A longitudinal retrospective observational study.

**Subjects:**

The study included all 538 consecutive stroke patients treated in Satakunta County, Finland, between January 2021 and January 2022.

**Methods:**

Functional recovery was assessed using the modified Rankin Scale (mRS), WHODAS 2.0, and HRQoL using the EQ-5D. Outcomes were evaluated at 1, 3, 6, and 12 months. Patients were categorized into 3 groups: no rehabilitation, primary care (PC) rehabilitation, and multidisciplinary (MD) rehabilitation. Longitudinal changes in mRS, WHODAS, and EQ-5D were further assessed at a 3-year follow-up.

**Results:**

MD rehabilitation resulted in significantly greater improvements in HRQoL and functional recovery compared with PC rehabilitation and no rehabilitation. Female patients reported lower HRQoL and poorer functioning than males; however, rates of improvement were similar between the sexes. Dependency (mRS > 2) was associated with lower HRQoL, although changes over the 1-year follow-up were comparable between dependent and independent patients. Depression emerged as the strongest predictor of HRQoL improvements. Substantial correlations were observed among mRS, EQ-5D, and WHODAS 2.0 scores, with the strength of these correlations increasing over time. At the 3-year follow-up, stroke survivors continued to exhibit decreasing HRQoL and functional status.

**Conclusions:**

Multidisciplinary rehabilitation substantially enhances functional recovery and HRQoL during the first year after stroke compared with primary care or no rehabilitation. Although women and patients with greater dependency report lower HRQoL, their rates of improvement are similar to those of other groups. Depression is a key determinant of HRQoL gains, underscoring the importance of integrating mental health support into rehabilitation pathways. The persistently low HRQoL observed 3 years post-stroke highlights the long-term burden of stroke and the need for sustained, comprehensive follow-up and rehabilitation strategies to address ongoing functional limitations.

## LAY ABSTRACT

- Functioning and Health-Related Quality of Life (HRQoL) improved significantly more in a multidisciplinary rehabilitation setting compared with primary care rehabilitation and no rehabilitation. Improvements were measured using the modified Rankin Scale (mRS), EQ-5D, and WHODAS 2.0.
- The mRS, WHODAS 2.0, and EQ-5D showed substantial correlation with one another. Correlations were weakest at the 3-month follow-up but improved significantly over time.
- Depression was the strongest predictor of changes in HRQoL. Addressing and managing depression is critical to improving quality of life after a stroke.
- Subacute and first-year stroke follow-up should be centred on multidisciplinary rehabilitation units, which prioritize addressing health-related quality of life and life satisfaction in their care models.

Stroke is a major cause of disability worldwide, causing billions of euros in direct and indirect costs to the community. Motor impairment, typically affecting movement of the face, arm, and leg on 1 side of the body, affects about 80% of stroke survivors (1). Cognitive impairment, with or without dementia, is observed in about 25–40% of stroke patients (2, 3), and approximately one-third of stroke survivors experience aphasia (4). About one-third of patients suffer from some type of mood disorder after stroke (5). These impairments significantly affect quality of life after stroke. However, depression has been reported to show a stronger correlation with quality of life compared with motor disability (6). Mood disorders are estimated to occur in up to 50% of patients during the acute phase and in over 20% during the subacute phase of stroke (7, 8), and they are suggested to decrease the quality of life, especially in older-onset stroke patients (9).

While post-stroke inpatient multidisciplinary rehabilitation has been shown to be beneficial and improve the functioning of stroke patients (10), outpatient rehabilitation standards are not clearly defined, and little is known about the effects of different programmes on health-related quality of life after stroke. The availability of post-stroke outpatient rehabilitation and follow-up is largely insufficient and unequal (11, 12), and the health-related quality of life in Finnish stroke survivors remains unknown.

Health-related quality of life (HRQOL) scores, which usually range from 0 (death) to 1 (full health), are frequently used to assess the level of HRQOL (13). These scores are reported to range between 0.47 and 0.68 after stroke (13,14) and between 0.45 and 0.59 according to the Swedish national stroke rehabilitation register (svereh.registercentrum.se). HRQOL after stroke has been shown to correlate with several factors, including functional constraints, age, sex, socioeconomic status, depression, and coping strategies (14–18).

This longitudinal retrospective clinical study evaluated functioning and health-related quality of life (HRQoL) over a 3-year period following stroke in Satakunta County, Finland. Functioning and HRQoL were assessed using WHODAS 2.0 (19–21), EQ-5D-3L (22), and the modified Rankin Scale (mRS) (23, 24). The primary objective was to determine whether differences in the implementation of stroke rehabilitation between specialized medical care and primary health care units resulted in differential outcomes in functioning and HRQoL during the first year after stroke. A secondary objective was to evaluate correlations between the assessment instruments, with particular emphasis on validating WHODAS 2.0 as a reliable tool for the longitudinal evaluation of HRQoL in stroke patients. Additionally, the study aimed to assess levels of functioning and HRQoL 3 years after stroke among patients in Satakunta County.

## MATERIALS AND METHODS

### Study design and participants

**Fig. 1:**
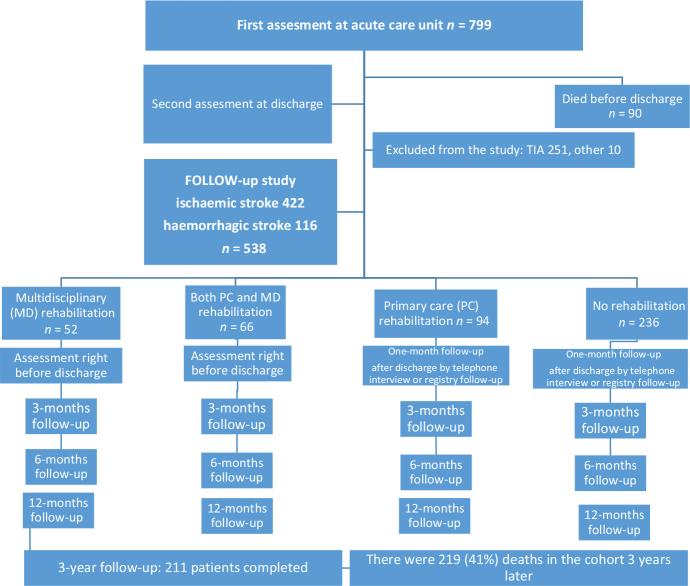
Flowchart of study design and participants.

This longitudinal retrospective observational study involved all patients diagnosed with stroke (ICD I60-I64, G45) between 18 January 2021, and 17 January 2022, in the Satakunta district, Finland (Fig. 1). The population of Satakunta 2021 was 214,281, which is 3.9% of the total population of Finland. The initial cohort comprised 847 consecutive patients. After excluding individuals diagnosed with transient global amnesia (G45.4) and those with vascular disease confined to the spinal region without cerebral involvement, 799 patients remained. Of these 799 patients, 422 were diagnosed with ischaemic stroke and 116 with intracranial haemorrhage (intracerebral or subarachnoid haemorrhage). Patients diagnosed with transient ischemic attack (*n* = 251), along with 10 individuals with uncertain or suspected vascular events not confirmed by a neurologist, were excluded. This resulted in a final cohort of 538 patients eligible for follow-up.

Among the 538 patients, 90 died during the acute phase of care (24 in acute care hospitals and 66 in primary healthcare centres). The mRS was successfully assessed at least once during the first year for 535 patients. A total of 270 participants completed the HRQoL questionnaire. At the 3-year follow-up – conducted through telephone interviews and review of medical records – there were 219 deaths (41%) within the original cohort of 538 individuals. A total of 211 participants (66% of survivors) completed the survey; however, 2 were unable to finish and their interviews were discontinued. Ninety-four individuals declined participation or were unable to take part due to aphasia, hearing impairment, cognitive decline (as reported by relatives or caregivers), or other unspecified reasons*.*

### Ethical considerations

Data collection adhered to European data protection regulations. Data were obtained from the unit’s Quality Registry Database (THL/2182/5.09.00/2019) and Satakunta County regional patient record system. Informed consent for follow-up was obtained from all individuals included in this study, who voluntarily participated in the 1- and 3-year assessments. All research involving human subjects complied with relevant national regulations, institutional policies, and the tenets of the Declaration of Helsinki (as amended in 2013). Ethical approval was granted by the Regional Ethics Committee, Satakunnan Sairaanhoitopiiri (SATSHP/697/13.01/2020).

### Baseline and outcome assessment

Changes in functioning and health-related quality of life (HRQoL) during the first year after stroke were assessed using standardized outcome measures. Functioning was assessed using the mRS at 6 time points: hospital admission, discharge, 1 month after discharge, and at 3, 6, and 12 months post-diagnosis. We analysed both the dichotomous classification (mRS 0–2 vs 3–6) and the full ordinal distribution of mRS scores, as the ordinal analysis offers greater statistical power when treatment effects are distributed across the entire range of disability (23, 24). HRQoL was assessed using the EQ-5D-3L (22) and WHODAS 2.0 (19–21) at 4 time points within the first year. A further follow-up was conducted approximately 3 years after stroke via telephone interview, during which EQ-5D-3L, the short version of WHODAS 2.0, and mRS were collected.

Because hospitalization duration varied and registry data had limitations, follow-up schedules were adapted across patient groups. The first follow-up (1 month) occurred within 1 month of discharge from acute care (mean hospital stay: 5 days) or on discharge for patients undergoing multidisciplinary (MD) rehabilitation (mean rehabilitation duration: 31 days). The second follow-up (3 months) took place 3 months after diagnosis for patients discharged directly home, or 1 month after discharge for those completing rehabilitation programmes. Subsequent assessments occurred at 6 and 12 months post-diagnosis, and the final follow-up was completed at approximately 3 years post-stroke via telephone interview (see Fig. 1).

Patients receiving rehabilitation in the Central Hospital Rehabilitation Unit (MD group) followed individualized rehabilitation plans with defined goals during the first year. For patients not participating in MD follow-up, stroke-specialized nurses conducted telephone interviews. Individuals unable or unwilling to participate were followed through registry data from the regional patient record system, which included mRS but lacked HRQoL measures. Eligibility criteria for interviews included age ≥ 18 years, proficiency in Finnish, adequate cognitive and linguistic ability, and willingness to participate. Rehabilitation procedures were categorized into 3 groups based on outpatient rehabilitation plans:

1. Multidisciplinary (MD) rehabilitation in the Central Hospital Unit.
2. Primary care (PC) rehabilitation with single therapies.
3. No rehabilitation.

Some overlap occurred between MD and PC rehabilitation participation. Patients in the MD rehabilitation group (*n* = 118) completed the West Coast Quality Registry questionnaire, which included the Hospital Anxiety and Depression Scale (25), the 15D instrument (25), and assessments on discharge as well as at 1, 6, and 12 months after discharge.

### Outcome

*Functioning.* Functioning was assessed using the modified mRS, which ranges from 0 to 6, where 0 indicates full independence and 6 represents death (23, 24). For analytical purposes, patients were also categorized into 2 groups: independent (mRS 0–2) and dependent (mRS 3–5).

*Health-Related Quality of Life (HRQoL).* HRQoL was assessed using the EQ-5D-3L, indexed according to Finnish value sets (22), where scores range from –0.011 (worst outcome) to 1 (best outcome), and the short version of WHODAS 2.0 (19–21). WHODAS 2.0 has demonstrated strong validity, reliability, and responsiveness across diverse medical conditions (20) and correlates well with established stroke outcome measures such as the mRS, NIHSS, and the Functional Independence Measure (FIM) (27–30). While most WHODAS research in stroke populations has been cross-sectional (27–30), the present study applied a longitudinal design. To enhance interpretability, WHODAS scores were converted to a 0–1 index scale, where 1 indicates no functional limitation and 0 indicates an extreme problem. Changes across 6 WHODAS subdomains – cognition, mobility, self-care, life activities, participation, and getting along – were also examined (19).

### Analysis of rehabilitation outcomes

Longitudinal changes in functioning and HRQoL were examined across the follow-up period. Changes in mRS scores were evaluated from hospital discharge to the 12-month follow-up. In the MD rehabilitation group, changes in WHODAS 2.0, EQ-5D, 15D, ICF, and HADS scores were analysed between the 3-month and 12-month assessments. Differences in HRQoL and functioning were further compared by sex (men vs women) and by age group, contrasting younger stroke survivors (≤ 62 years) with older individuals.

### Statistical analysis

All statistical analyses were performed using IBM SPSS (IBM Corp, Armonk, NY, USA). Wilcoxon matched-pairs signed-rank tests were applied to compare the magnitude and direction of differences between paired score distributions, and independent-samples Mann–Whitney U tests were used for post hoc analyses. Statistical significance was defined as a *p*-value < 0.05*.* Correlation analyses were conducted to examine relationships between ICF core set subdomains and EQ-5D scores. Summary variables representing different aspects of functioning were computed, with careful verification of their comparability, and associations were assessed using Spearman’s rho (*r*_s_). Agreement coefficients were interpreted as follows: 0–0.20 = poor, 0.21–0.40 = fair, 0.41–0.60 = moderate, 0.61–0.80 = substantial, and 0.81–1.0 = near-perfect (31). Only correlations above 0.40 were considered significant for reporting. A stepwise linear regression model was used to identify predictors of changes and to identify the ICF subdomains that best predicted HRQoL, as measured by EQ-5D, at the 1-year and 3-year follow-up assessments.

## RESULTS

### Baseline characteristics

General characteristics are presented in Table I. A significant difference in mean age was observed between men and women (mean difference 5.6 years; 95% CI, 3.4–7.7; *p* < 0.001). Among the 538 patients, 22% (*n* = 118) participated in multidisciplinary (MD) rehabilitation; 28% (*n* = 152) had no rehabilitation, and 34 received primary care (PC) therapies only but were engaged through phone contact; and 50% (*n* = 268) contributed data solely through the regional patient record system.

**Table I:** Descriptive variables at baseline

Variable	No. (%)
Sex (male)	254 (47%)
Age (mean) Age (men) Age (women)	74.6 (SD = 12.8) 71.7 (SD = 12.0) 77.3 (SD = 12.9)
Diagnosis Ischaemic stroke Haemorrhagic stroke	422 (78%) 116 (22%)
Discharge after acute care Community hospital rehabilitation unit Home Specialized rehabilitation unit General ward Died Other	170 (32%) 149 (28%) 94 (18%) 80 (15%) 24 (4%) 21 (3%)
1 year follow-up Living at home Independently Assisted Nursing home Other Died	265 (49%) 82 (15%) 53 (10%) 2 (0.2%) 136 (25%)
New vascular event under first year Other health complication	40 (7%) 21 (4%)
Rehabilitation under first year after acute care No rehabilitation Specialized rehabilitation unit outpatient	236 (48%) 118 (22%)
PC outpatient rehabilitation Physiotherapy Occupational therapy Speech therapy Home-based therapy Other	161 (30%) 141 48 15 114 51
mRS on discharge from hospital Independent (0–2) Dependent (3–5) Died (6)	258 (48%) 190 (35%) 90 (17%)
mRS 1-year follow-up Independent (0–2) Dependent (3–5) Died (6) Missing	263 (49%) 137 (26%) 135 (25%) 3

The longitudinal HRQoL analysis included 203 patients who completed the WHODAS 2.0 and 217 who completed the EQ-5D. Among EQ-5D respondents, 80% (*n* = 173) were classified as independent and 20% (*n* = 44) as dependent. Similarly, 80% (*n* = 162) of WHODAS 2.0 respondents were independent and 20% (*n* = 41) were dependent.

### Modified Rankin Scale (mRS)

**Fig. 2:**
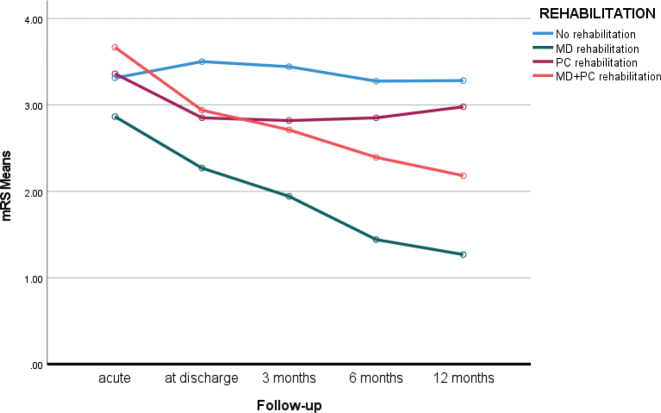
Changes in mRS (0–6 with 0 indicating no symptoms and 6 indicating death) in different rehabilitation groups at 1-year follow-up: No rehabilitation, Multidisciplinary (MD) outpatient rehabilitation, primary care rehabilitation (PC), and primary care MD combination.

Significant improvements in mRS scores were observed across assessment points, except between 6 and 12 months (Table II, Fig. 2). Patients receiving multidisciplinary (MD) rehabilitation showed significant improvement up to 6 months, with no further change after 12 months. The primary care (PC) rehabilitation group reached a plateau after 3 months. Mean changes in mRS scores from discharge to 12 months were as follows: no rehabilitation group 0.2 (SD = 1.3), multidisciplinary (MD) rehabilitation: 1.0 (SD = 1.0), combined MD and primary care group: 0.8 (SD = 1.0) and primary care rehabilitation (N = 94): –0.13 (SD = 1.2). Independent-samples Mann-Whitney *U post hoc* analyses revealed significant differences between groups: MD vs no rehabilitation or PC rehabilitation (mean difference –0.30, Z = –6.38, *p* < 0.001); MD vs PC rehabilitation (mean difference –0.45, Z = –4.75, *p* < 0.001).

**Table II:** . Changes in functioning as measured by the mRS (scale 0–6, where 0 indicates no symptoms and 6 indicates death) are indicated for all patients, those in multidisciplinary rehabilitation units, and those in primary care units at different time points: admission, discharge, 3-month follow-up, 6-month follow-up, and 12-month follow-up

Factor	Mean	*n*	SD	Standard error	Z	Asymptotic	Sig. 2-sided
All patients							
Admission Discharge	3.3	538	1.2	1,097	–3.04		0.002
	3.2	538	1.6				
Discharge 3 months	3.2	538	1.6	367	–4.38		< 0.001
	3.1	538	1.7				
3 months 6 months	3.1	536	1.7	696	–5.10		< 0.001
	2.9	536	2.1				
6 months 12 months	2.9	535	2.1	410	–1.23		NS
	2.9	535	2.2				
Multidisciplinary rehabilitation unit							
Admission Discharge	3.3	118	1.0	137	–7.35		< 0.001
	2.6	118	0.8				
Discharge 3 months	2.6	118	0.8	56.9	–4.72		< 0.001
	2.4	118	1.0				
3 months 6 months	2.4	118	1.0	73.3	–5.32		< 0.001
	2.0	118	1.2				
6 months 12 months	2.0	118	1.2	53.5	–3.77		< 0.001
	1.8	118	1.4				
Primary care rehabilitation							
Admission Discharge	3.4	174	1.0	201	–7.96		< 0.001
	2.8	174	0.9				
Discharge 3 months	2.8	174	0.9	81	–2.76		0.006
	2.7	174	1.0				
3 months 6 months	2.7	173	1.0	115	–1.64		NS
	2.6	173	1.3				
6 months 12 months	2.6	174	1.3	88.2	–0.15		NS
	2.6	174	1.5				

### Age and gender

Younger patients (< 62 years) had lower mRS scores and demonstrated greater improvement at 12 months compared with older patients (≥ 62 years) (mean difference 0.59, Z = –2.75, *p* = 0.006). Women consistently exhibited higher (worse) mRS scores than men (e.g., on discharge: mean difference –0.35, Z = 2.69, *p* = 0.007). However, no significant gender differences were found in the magnitude of improvement over the 1-year follow-up period.

### EQ-5D

**Fig. 3:**
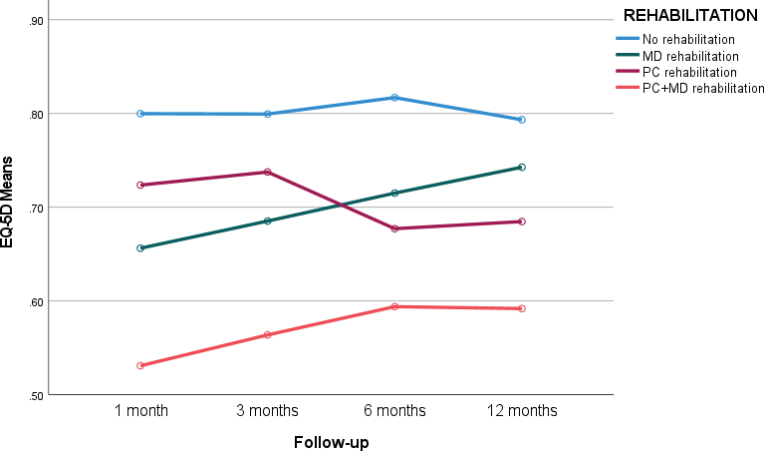
EQ-5D index (from –0.011 to 1 with 1 indicating full health) under 1-year follow-up in different rehabilitation groups: No rehabilitation, Multidisciplinary (MD) outpatient rehabilitation, primary care rehabilitation (PC), and primary care MD combination.

No significant overall improvement in EQ-5D scores was observed during the first year (Table III). However, within the multidisciplinary (MD) rehabilitation group, HRQoL improved significantly during the first 6 months. Between-group differences are shown in Fig. 3. Significant differences were identified between the MD group and the other groups (mean difference 0.13, Z = 3.68, *p* < 0.001). Changes in EQ-5D were for no rehabilitation: Mean decline –0.01 (SD = 0.16); PC rehabilitation: Mean decline –0.05 (SD = 0.15); MD rehabilitation: Mean improvement 0.08 (SD = 0.15); Combined MD and PC: Mean improvement 0.04 (SD = 0.19).

**Table III:** Health Related Quality of Life (HRQoL) as measured with the EQ-5D (with lowest HRQoL index –0.011 and 1 meaning full health) for all patients, for multidisciplinary rehabilitation unit and primary care rehabilitation 1-year follow-up. Index at 1 month, 3-month follow-up, 6-month follow-up, 12-month follow-up, and 3-year follow-up

Factor	Mean	*n*	SD	Standard error	Z	Asymptotic	Sig. 2-sided
All patients							
1 month 3 months	0.70	232	0.23	440	1.12		NS
	0.72	239	0.21				
3 months 6 months	0.72	239	0.21	465	1.57		NS
	0.73	237	0.21				
6 months 12 months	0.73	237	0.22	405	0.83		NS
	0.72	233	0.21				
3 months	0.77	153	0.20	150	–4.76		< 0.001
3 years	0.63	183	0.25				
12 months	0.77	123	0.19	136	–3.80		< 0.001
3 years	0.63	183	0.25				
Multidisciplinary rehabilitation unit							
1 month 3 months	0.59	86	0.24	136	1.46		NS
	0.62	96	0.21				
3 months 6 months	0.62	96	0.21	156	2.35		0.019
	0.65	102	0.23				
6 months 12 months	0.65	101	0.23	132	1.15		NS
	0.66	101	0.24				
Primary care rehabilitation							
1 month 3 months	0.57	87	0.23	135	1.10		NS
	0.62	87	0.22				
3 months 6 months	0.62	90	0.23	143	1.01		NS
	0.60	86	0.22				
6 months 12 months	0.60	86	0.22	126	0.20		NS
	0.60	85	0.21				

Age had no significant effect on EQ-5D outcomes. Women reported lower EQ-5D scores at both the 3-month and 6-month follow-ups (mean difference 0.06, Z = –2.35, *p* = 0.03), although this difference was no longer significant at the 12-month follow-up (Z = 1.23, *p* = ns).

### WHODAS 2.0

**Fig. 4:**
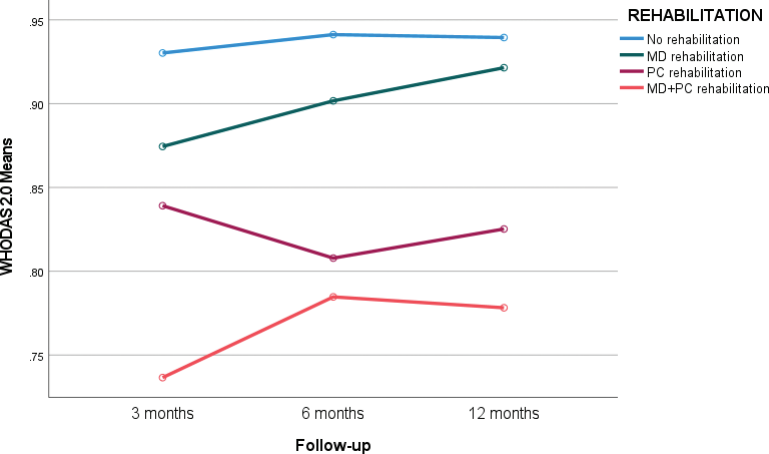
Changes in WHODAS 2.0 index (from 0 to 1 with 1 indicating full health) under 1-year follow-up in different rehabilitation groups: No rehabilitation, Multidisciplinary (MD) outpatient rehabilitation, primary care rehabilitation (PC), and primary care MD combination.

Significant improvements in WHODAS 2.0 scores were observed up to 6 months (Table IV), with the largest change occurring between 3 and 6 months (mean difference 0.02, Z = 3.16, *p* = 0.002).Changes in WHODAS scores by rehabilitation group are shown in Fig. 4. Among patients receiving multidisciplinary (MD) rehabilitation, improvement was observed between 3 and 6 months (mean difference 0.04, Z = 3.36, *p* < 0.001). In the group without rehabilitation, a mean improvement of 0.02 (Z = 2.54, *p* = 0.01) was also noted during this period. For the primary care (PC) rehabilitation group, the mean improvement was 0.02 (Z = 2.66, *p* = 0.008). By 12 months, the mean difference from 3 months to 12 months was 0.04 for the MD group (Z = 2.19, *p* = 0.03), 0.02 for the PC group (Z = –0.40, *p* = ns), and 0.01 for the no-rehabilitation group (Z = 1.49, *p* = ns).

**Table IV:** WHODAS 2.0 index (from 0 to 1, with 1 indicating full health) under 1-year follow-up for all patients, for multidisciplinary rehabilitation unit and primary care rehabilitation. Assessments at 1 month, 3-month, 6-month, and 12-month follow-up

Factor	Mean	*n*	SD	Mean difference	Z	Asymptotic	Sig. 2-sided
All patients							
1 month 3 months	0.89	149	0.14	254	1.64		NS
	0.86	220	0.16				
3 months 6 months	0.86	220	0.16	530	3.16		0.002
	0.88	234	0.15				
6 months 12 months	0.88	234	0.15	518	0.44		NS
	0.89	230	0.15				
3 months	0.89	150	0.15	196	–5.71		< 0.001
3 years	0.76	177	0.24				
12 months	0.91	124	0.13	171	–6.21		< 0.001
3 years	0.76	177	0.24				
Multidisciplinary rehabilitation unit							
1 month 3 months	0.61	3	0.41	1.12	1.34		NS
	0.80	95	0.18				
3 months 6 months	0.80	95	0.18	193	3.47		< 0.001
	0.84	99	0.16				
6 months 12 months	0.84	99	0.16	185	0.64		NS
	0.84	99	0.18				
Primary care rehabilitation							
1 month 3 months	0.84	37	0.14	34.7	–1.41		NS
	0.82	28	0.16				
3 months 6 months	0.82	28	0.16	26.5	–1.89		NS
	0.83	31	0.18				
6 months 12 months	0.83	31	0.18	34.7	0.16		NS
	0.83	29	0.15				

Subdomain analyses indicated significant improvements in cognition, getting along, and life activities, but no significant changes in mobility, self-care, or participation (Table V).

**Table V:** Changes in functioning as measured with WHODAS 2.0 6 subdomains (from 0 to 1, with 1 indicating full function) for all patients regardless of rehabilitation process at 1-year follow-up

Factor	Mean	*n*	SD	Mean difference	Z	Asymptotic	Sig. 2-sided
Life activity 3 months Life activity 12 months	0.81	239	0.27	278	2,67		0.008
	0.85	230	0.25				
Cognition 3 months Cognition 12 months	0.88	220	0.18	229	2.11		0.03
	0.90	230	0.16				
Getting along 3 months Getting along 12 months	0.94	239	0.16	95.1	2.13		0.03
	0.96	230	0.13				
Self-care 3 months Self-care 12 months	0.92	239	0.17	108	1.36		NS
	0.93	230	0.16				
Participation 3 months Participation 12 months	0.83	239	0.21	351	0.63		NS
	0.85	230	0.18				
Mobility 3 months Mobility 12 months	0.81 0.81	239 230	0.27 0.26	316	–0.27		NS

### Gender and age

Women consistently reported lower WHODAS scores at all assessment points: at the first follow-up (Z = –2.32, *p* = 0.02), 3 months (Z = –2.34, *p* = 0.02), 6 months (Z = –2.22, *p* = 0.03), and 12 months (Z = –2.62, *p* = 0.009). No significant age-related differences were identified.

### Correlations between mRS, WHODAS 2.0, and EQ-5D

Moderate to substantial correlations were found between mRS and WHODAS at all time points (e.g., 6 months: *r* = –0.68, *p* < 0.001). Substantial correlations were also observed between mRS and EQ-5D at 6 and 12 months (e.g., *r* = –0.61, *p* < 0.001). WHODAS 2.0 and EQ-5D showed substantial correlations throughout the follow-up period (e.g., 3 months: *r* = 0.71, *p* < 0.001). Changes in EQ-5D from 3 to 12 months correlated moderately with changes in WHODAS (r = 0.41, *p* < 0.001). MRS change was not correlated with WHODAS and EQ-5D changes. Correlations for changes over the 3–12-month interval are presented in Table VI. Changes in HADS depression scores correlated significantly with changes in EQ-5D (*r* = 0.36, *p* < 0.001) and WHODAS 2.0 (*r* = 0.48, *p* < 0.001).

**Table VI:** Correlation coefficients between changes from 3-month to 12-month follow-up in functioning measured with mRS, EQ-5D, WHODAS 2.0, 15D, HADS (anxiety and depression). and age

Factor	Change in mRS	Change in EQ-5D	Change in WHODAS 2.0	Change in 15D ICF
Change in WHODAS 2.0	NS	0.41	–	0.55
Change in 15D ICF	NS	0.52	0.56	–
Change in HADS anxiety	NS	NS	NS	0.29
Change in HADS depression	NS	0.36	0.48	0.44
Age	–0.39	NS	NS	NS

### Predictors of change

The only significant predictor of change in EQ-5D was change in depressive symptoms. A 1-point decrease in HADS depression score was associated with a 0.02-point increase in EQ-5D (R² = 0.208, *p* < 0.001). Similarly, the strongest predictor of change in WHODAS 2.0 was change in depression; a 1-point reduction in HADS depression corresponded to a 0.03-point improvement in WHODAS (R² = 0.321, *p* < 0.001). Change in mRS was correlated only with age; however, age showed no association with changes in EQ-5D, WHODAS 2.0, 15D, ICF, or HADS outcomes (see Table VI).

### Specialized rehabilitation outcomes for 15D index and HADS

Among patients who participated in specialized multidisciplinary rehabilitation (MD), the mean 15D index at discharge was 0.86. By the 3-month follow-up, the index had decreased to 0.83, representing a mean decline of 0.03 (95% CI 0.01–0.04; *p* = 0.009). Between the 3-month and 6-month follow-ups, the 15D index increased, with a mean improvement of 0.02 (95% CI 0.00–0.04; *p* = 0.05). No significant changes were observed between the 6-month and 12-month follow-ups.

No significant changes were detected in HADS anxiety scores across the follow-up period (two-way analysis [Asymptotic Sig. (2-sided test)] *p* = 0.37). In contrast, HADS depression scores increased significantly between discharge and the 3-month follow-up, with a mean rise of 1.3 points (Asymptotic Sig. [2-sided test] *p* = 0.014).

### Dependency at 3-month follow-up and HRQoL

Significant associations were observed between dependency (mRS >2) and lower HRQoL. The mean EQ-5D difference between independent and dependent patients was 0.28 at the 3-month follow-up (Z = –5.58, *p* < 0.001) and 0.30 at the 12-month follow-up (Z = –6.10, *p* < 0.001). For WHODAS 2.0, the mean difference between independent and dependent patients was 0.18 at 3 months (Z = –6.37, *p* < 0.001) and increased to 0.25 at 12 months (Z = –6.34, *p* < 0.001).

Although dependent patients consistently had lower HRQoL scores at all follow-ups, the magnitude of HRQoL change from 3 to 12 months did not differ significantly between groups (EQ-5D: Z = 0.41, *p* = ns; WHODAS: Z = 1.0, *p* = ns). Changes in anxiety and depression scores were not associated with age or sex.

### Functional recovery (mRS)

Independent patients demonstrated significantly greater improvement in mRS scores over the 1-year follow-up period compared with dependent patients. The mean difference between groups at 12 months was –0.74 points (Z = 8.01, *p* < 0.001).

### 3-year follow-up

**Fig. 5:**
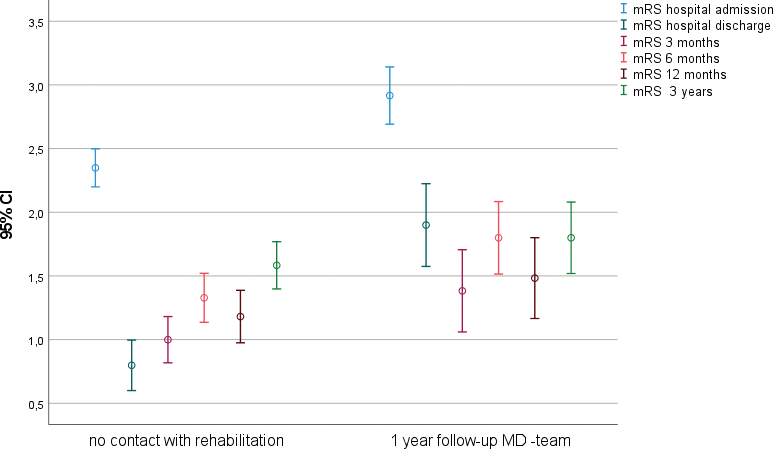
Changes in mRS under 3-year follow-up for patients without contact medical rehabilitation and patients attending MD follow-up under the first year: mRS hospital admission, mRS hospital discharge, mRS 3 months, mRS 6 months, mRS 1 year, and mRS 3 years.

**Fig. 6:**
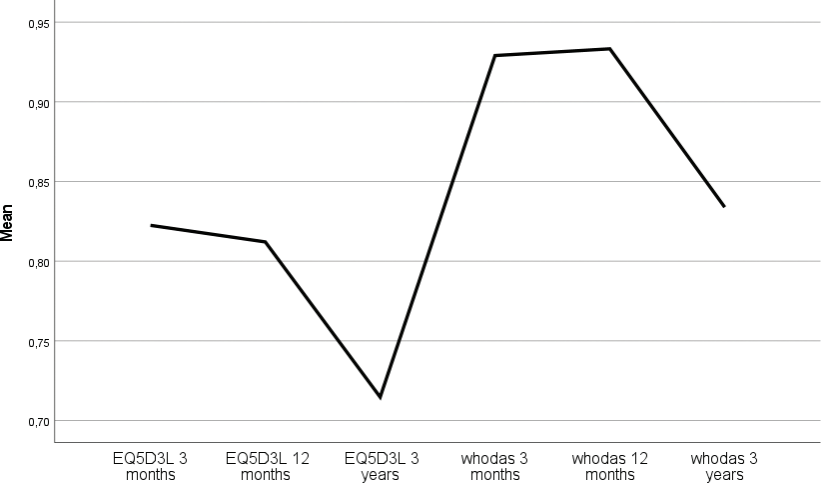
Changes in HRQoL under 3-year follow-up for all patients after stroke: EQ5D after 3 months, EQ5D 12 months, EQ5D 3 years, WHODAS index 3 months, WHODAS index 12 months, WHODAS index 3 years.

At the 3-year follow-up, conducted through telephone interviews and review of clinical records, 219 of the 538 individuals (41%) had died. The results are presented in Figs 5 and 6, demonstrating significantly lower HRQoL and reduced functioning at 3 years. The EQ-5D index declined from 0.78 (SD = 0.20) at the 1-year follow-up to 0.63 (SD = 0.26) at the 3-year follow-up (Z = –3.80, *p* < 0.001). The WHODAS 2.0 index similarly decreased from 0.92 (SD = 0.13) at 1 year to 0.76 (SD = 0.24) at 3 years (Z = –6.21, *p* < 0.001). Changes in mRS scores are shown in Fig. 5, indicating an increase from 1.27 (SD = 1.3) at 1 year to 1.64 (SD = 1.1) at 3 years (Z = 5.35, *p* < 0.001).

The mean age of survivors at the 3-year follow-up was 68.6 years (SD = 12.0), compared with an initial mean age of 74.6 years (SD = 12.8) for the total cohort. Survivors exhibited better functional status (mRS) throughout follow-up compared with the entire post-stroke cohort: at 3 months (1.1 vs 3.1), at 6 months (1.5 vs 2.9), and at 12 months (1.3 vs 2.9). Correspondingly, EQ-5D index scores were higher among survivors at both 3 months (0.78 vs 0.72) and 12 months (0.78 vs 0.72).

At the 3-year follow-up, EQ-5D index scores were significantly associated with 3 factors: mRS, emotional impact (“being emotionally affected”), and difficulty maintaining posture (“standing for long periods”) (R² = 0.71, *p* < 0.001). At the 1-year follow-up, the variables that predicted HRQoL (EQ-5D index) at 3 years were EQ-5D index, difficulty “walking a long distance”, and difficulty “getting dressed”. Substantial correlations at 3-year follow-up were observed between mRS, WHODAS 2.0, and EQ-5D (*r* = –79, *p* < 0.001, vs *r* = –0.82, *p* < 0.001). WHODAS 2.0 and EQ-5D showed substantial correlations (*r* = 0.80, *p* < 0.001).

## DISCUSSION

In this longitudinal retrospective study, we found that modest improvements in mRS scores can still occur beyond 6 months after stroke, but only among patients evaluated in a specialized multidisciplinary rehabilitation unit. The most substantial functional gains were observed within the first 6 months, a period during which spontaneous recovery likely plays a major role. Functional status measured by the mRS at the 12-month follow-up strongly predicted both functioning and HRQoL at 3 years post-stroke. The 7-level mRS offers several important strengths. It captures the full continuum of post-stroke functional outcomes – from no symptoms to death – and its categories are intuitive for both clinicians and patients. The mRS demonstrates strong concurrent validity through robust associations with stroke severity indicators, such as infarct volume, as well as with other validated stroke outcome measures (32). It has also consistently distinguished effective from ineffective acute stroke therapies in large, well-powered clinical trials (32). Although its relatively coarse categorization may reduce sensitivity to subtle functional changes compared with more granular instruments, even a 1-point change on the mRS is widely regarded as clinically meaningful (32). A growing body of evidence supports analysing the full ordinal distribution of the mRS rather than relying on dichotomized outcomes. Ordinal analysis offers greater statistical power, particularly when treatment effects span multiple outcome levels and include both favourable and unfavourable categories – such as death or symptomatic haemorrhage (33–35). This approach is particularly advantageous when treatment effects are not concentrated at one end of the scale but distributed across several functional categories (36).

### Rehabilitation and quality of life

Our findings indicate that patients who did not receive multidisciplinary follow-up showed no improvement in HRQoL as measured by the EQ-5D. In contrast, those who participated in multidisciplinary rehabilitation demonstrated both statistically and clinically significant gains, with a mean EQ-5D improvement of 0.08. Kim et al. have reported that clinically meaningful changes in EQ-5D in stroke populations range from 0.08 to 0.12 (37), suggesting that the improvements observed in our cohort were indeed of clinical relevance. Changes in EQ-5D were most strongly correlated with changes in anxiety and depression, as well as with changes in the multidimensional 15D index (26).

The strongest correlations between changes in WHODAS scores and patient outcomes were seen with depression and the 15D index, underscoring the importance of recognizing and managing depressive symptoms during the first year of stroke rehabilitation (38).

At the 3-year follow-up, we observed significantly lower HRQoL scores – reflected in the EQ-5D index, EQ-VAS, and WHODAS – compared with both the 3-month and 1-year assessments. This decline highlights the persistence of long-term challenges and the need for ongoing monitoring and support among stroke survivors.

### Depression, anxiety, and disability

We found that lower HRQoL was associated with greater levels of depression, anxiety, and disability, in line with previous research (38–40). However, unlike earlier studies (38, 39, 41), we did not observe a significant correlation between age and HRQoL. Being female was associated with lower EQ-5D scores at the 3-month follow-up and lower WHODAS scores at both the 3- and 12-month assessments. Older age and female sex were also associated with worse functional status as reflected by higher mRS scores; however, the magnitude of mRS improvement over time was similar for both men and women. This unexpected association between sex and mRS is likely attributable to the higher mean age of women in our cohort compared with men.

### Role of depression in HRQoL

Improvements in HADS depression scores were the strongest predictors of HRQoL. This is consistent with the meta-analysis by Ayerbe et al. (42), which reported that post-stroke depression is closely associated with lower quality of life. Our findings highlight the critical importance of identifying and treating depression to optimize HRQoL during the first year after stroke. The role of depression and other mood disorders in stroke survivors should be explicitly acknowledged, and greater efforts are needed to provide timely and effective treatment to enhance rehabilitation outcomes. In specialized rehabilitation units, mood disorders are systematically screened, and treatment protocols commonly include the use of selective serotonin reuptake inhibitors (SSRIs), such as fluoxetine, in accordance with Finnish Clinical Practice Guidelines (www.kaypahoito.fi). In our study, 61% of patients in multidisciplinary rehabilitation received fluoxetine or other SSRI/SNRI medications, compared with only 18% of those who did not undergo rehabilitation or received only primary care rehabilitation. Patients in multidisciplinary rehabilitation also had access to psychological support, including at least 3 consultations with a psychologist during the first year, supplemented by individual or group-based therapeutic interventions. In contrast, psychological or neuropsychological services were not available in primary care rehabilitation programmes.

### Correlations among measures

The mRS, EQ-5D, and WHODAS 2.0 demonstrated moderate to substantial correlations, supporting their utility as complementary instruments for the longitudinal assessment of rehabilitation outcomes. Although WHODAS 2.0 is widely used across various medical conditions to evaluate disability and quality of life (19), only a limited number of longitudinal studies have applied it to stroke survivors during extended follow-up (27–30). Furthermore, studies specifically evaluating WHODAS as an outcome measure for stroke rehabilitation interventions remain scarce. To our knowledge, this is the first study to demonstrate a substantial correlation between WHODAS and EQ-5D in a stroke population, although such associations have been documented in other clinical conditions (43). In our cohort, WHODAS 2.0 showed a consistently strong correlation with EQ-5D across all measurement points (*r* = 0.69–0.71). WHODAS also appeared more sensitive than EQ-5D in detecting minor improvements during the first-year follow-up, regardless of rehabilitation type. In contrast, EQ-5D improvements were observed only in patients who completed multidisciplinary rehabilitation. Tarvonen-Schröder et al. (27) reported a moderate correlation between mRS and WHODAS 2.0 in subacute stroke on discharge (*r* = 0.49), comparable to our finding at the 3-month follow-up (*r* = –0.46). Hartley et al. (44) reported a weak correlation between EQ-5D and mRS at admission (*r* = –0.073) that strengthened to –0.362 on discharge; similarly, we observed a comparable association at 3 months (*r* = –0.39). Across measures, correlations between functioning and HRQoL were weaker at 3 months but strengthened by 12 months, likely reflecting evolving patient coping, adjustment, and greater awareness of functional limitations. Early after stroke, patients may hold optimistic or unrealistic expectations, potentially inflating perceived HRQoL during the initial months.

Analysis of WHODAS 2.0 subdomains showed the greatest improvements in life activities over 1 year, while no significant gains were observed in activities of daily living (ADL) or mobility. Among patients in specialized rehabilitation, the 15D ICF index initially declined from 0.86 on discharge to 0.83 at 3 months, before increasing to 0.85 at 6 months. Initial decreases in HRQoL have been described by Barbosa et al. (41), whereas Pucciarelli et al. (45) and Orman et al. (46) reported continuous improvements. In our study, EQ-5D and WHODAS 2.0 exhibited steady improvement during the first year, while the 15D index followed a decrease-then-increase pattern.

At the 3-year follow-up among stroke survivors, both functional status and HRQoL had significantly declined compared with the 3-month and 1-year assessments*.* This decline was particularly pronounced among patients who did not participate in rehabilitation services. Such marked deterioration suggests that many long-term consequences of stroke either persist or worsen over time (47). Multiple determinants may shape long-term HRQoL outcomes, including motor deficits (47, 48), dependence in activities of daily living (48, 49), depressive symptoms (47, 49–53), overall functional status (54), the severity of neurological impairment (54), the presence of aphasia (54), and the anatomical location of the stroke lesion (55).

### Strengths and limitations

This longitudinal, retrospective study is a part of a larger project that aims to develop rehabilitation practices resulting in higher health-related quality of life for all stroke patients in Finland. The strength of this study is its dimensions, while all patients willing to adequately answer the questions of quality of life and capable of doing so were participating. As a longitudinal study, it reflects the individual progress of patients and increases the knowledge of changes in functionality and quality of life within the first year after stoke. The limitation of this study is that we still do not have the data on patient-reported assessment of health-related quality of life of nearly half of stroke patients, who were not able or not willing to participate. The retrospective nature of data collection exposes it to reporting biases.

One of the limitations of our study was the use of the telephone interview at the 1- and 3-year follow up, which could have prompted inaccurate responses and introduced observer bias.

### Conclusions

This longitudinal study shows that modest functional improvements can still occur beyond 6 months after stroke, but only among patients receiving specialized multidisciplinary rehabilitation. Functional status at 12 months strongly predicted both functioning and HRQoL at 3 years. Multidisciplinary rehabilitation led to clinically meaningful gains in HRQoL, while no improvement was observed among patients without such follow-up. Depression emerged as the strongest predictor of HRQoL, highlighting the need for systematic identification and treatment of mood disorders. WHODAS 2.0, EQ-5D, and mRS demonstrated moderate to substantial correlations, supporting their combined use in long-term outcome assessment. By 3 years, both functioning and HRQoL declined significantly, particularly among patients who did not receive rehabilitation, indicating that many long-term consequences of stroke persist or worsen over time. These findings underscore the importance of sustained, specialized rehabilitation and continued monitoring of psychological well-being to optimize long-term recovery.
